# Evaluation of Academic Detailing Programme on Childhood Diarrhoea Management by Primary Healthcare Providers in Banke District of Nepal

**DOI:** 10.3329/jhpn.v31i2.16388

**Published:** 2013-06

**Authors:** Saval Khanal, Mohamed Izham b. Mohamed Ibrahim, Pathiyil Ravi Shankar, Subish Palaian, Pranaya Mishra

**Affiliations:** ^1^Department of Pharmacy, Sunsari Technical College, Dharan, Nepal;; ^2^College of Pharmacy, Qatar University, Doha, Qatar;; ^3^Department of Clinical Pharmacology and Therapeutics, KIST Medical College, Lalitpur, Nepal;; ^4^Department of Pharmacology, College of Medical Sciences, Bharatpur, Nepal;; ^5^Department of Pharmacology, Manipal College of Medical Sciences, Pokhara, Nepal

**Keywords:** Academic detailing, Developing countries, Diarrhoea, Oral rehydration solution, Primary healthcare, Zinc, Nepal

## Abstract

Academic detailing is rarely practised in developing countries. A randomized control trial on healthcare service was conducted to evaluate the impact of academic detailing programme on the adherence of primary healthcare providers in Banke district, Nepal, to childhood diarrhoea treatment guidelines recommended by World Health Organization/United Nations Children's Fund (WHO/UNICEF). The participants (N=209) were systematically divided into control and intervention groups. Four different academic detailing sessions on childhood diarrhoea management were given to participants in the intervention group. At baseline, 6% of the participants in the control and 8.3% in the intervention group were adhering to the treatment guidelines which significantly (p<0.05) increased among participants in the intervention (65.1%) than in the control group (16.0%) at the first follow-up. At the second follow-up, 69.7% of participants in the intervention group were adhering to the guidelines, which was significantly (p<0.05) greater than those in the control group (19.0%). Data also showed significant improvement in prescribing pattern of the participants in the intervention group compared to the control group. Therefore, academic detailing can be used for promoting adherence to treatment guidelines in developing countries, like Nepal.

## INTRODUCTION

Academic detailing, an educational intervention programme, in which a trained healthcare professional visits clinicians in their offices to provide evidence-based information, has been used as a major tool to improve rational use of medicines since it was started for the first time in 1983 (1,2). The process involves face-to-face education of clinicians by trained academic detailers. The academic detailers are usually healthcare professionals, like pharmacists, clinicians, nurses, or any persons trained on the subject matter (1). Academic detailing programmes have been particularly used for improving the knowledge of clinicians on particular topics, changing prescribing pattern of targeted drugs to be consistent with medical evidence, providing medical care in cost-effective ways, or minimizing risks to patients due to wrong practice (3-6). A meta-analysis, including 69 studies involving more than 15,000 healthcare professionals, reported that academic detailing, as a single intervention or in combination with other interventions, had relatively small but consistent and important influence on prescribing behaviour. The same study also suggested that effects of academic detailing on other types of professional performance of healthcare providers varied from small to modest improvements; however, the authors were unable to quantify such variations (7).

Academic detailing has been practised for many years in developed countries (2,8-10). However, the concept of academic detailing is still at premature phase in developing countries, like Nepal (11). There are very few studies on academic detailing from developing countries. Hence, our research was carried out in Banke district of Nepal to evaluate the impact of academic detailing programme on childhood diarrhoea management by primary healthcare providers. Prior to this research, a small pilot study was carried out on 10 healthcare professionals in the same district. The pilot study was done to ensure methodological validation, including training of academic detailers, data collectors, and simulated clients (12). This article aims to describe the feasibility of academic detailing in developing countries, like Nepal, by evaluating the outcome of the same programme in terms of (i) clinicians’ prescribing pattern, (ii) adherence to childhood diarrhoea treatment guidelines, and (iii) change in their prescription cost. The following hypotheses were set prior to this research:

(a) Academic detailing will significantly increase prescribing of the oral rehydration solutions and zinc for the treatment of diarrhoea without dehydration by primary healthcare providers in the intervention group compared to the control group.
(b) Academic detailing will significantly decrease the prescription of unnecessary medicines, like antimicrobials, medicines affecting gastric motility, vitamins, and enzyme preparations among participants in the intervention group to treat acute diarrhoea without dehydration compared to the control group.
(c) Academic detailing will significantly increase the compliance with WHO/UNICEF-endorsed “Diarrhoea treatment guidelines, including new recommendations for the use of ORS and zinc supplementation for clinic-based healthcare workers” among the primary healthcare providers in the intervention group compared to the control group.

## MATERIALS AND METHODS

### Study design

A randomized control trial on healthcare was conducted to evaluate the impact of academic detailing programme on the childhood diarrhoea management by primary healthcare providers in Banke district of Nepal. This study consisted of a phase of data collection prior to four different academic detailing sessions and two phases of data collections after those academic detailing sessions.

### Study site and duration

The study was carried out from July 2009 to March 2010 in Banke district of Nepal. Banke district lies in the Midwestern region of Nepal. It is a plain flat land adjacent to the Indian border. The health institutions in urban part of the district consist of a 300-bed government hospital, two 100-bed private hospitals, a 150-bed nursing home, and an 850-bed tertiary-care teaching hospital whereas the rural part consists of primary healthcare centres, health posts, and sub-health posts.

Both urban and rural parts of this district have private practitioners, especially those having their clinics inside some pharmacies. This district is known to be one of the susceptible districts for childhood diarrhoea, and the Ministry of Health of the Government of Nepal has started Control of Diarrhoeal Disease (CDD) programme under Community-based Management of Childhood Illness (CB-IMCI) project to combat the childhood diarrhoea in this district. Annual report published by the Department of Health Services, Ministry of Health, Government of Nepal, reported visit of a total of 4,662 diarrhoeal children (between 2 months and 5 years of age) in the government healthcare institutions of Banke district (13).

### Study population

**Figure 1.**
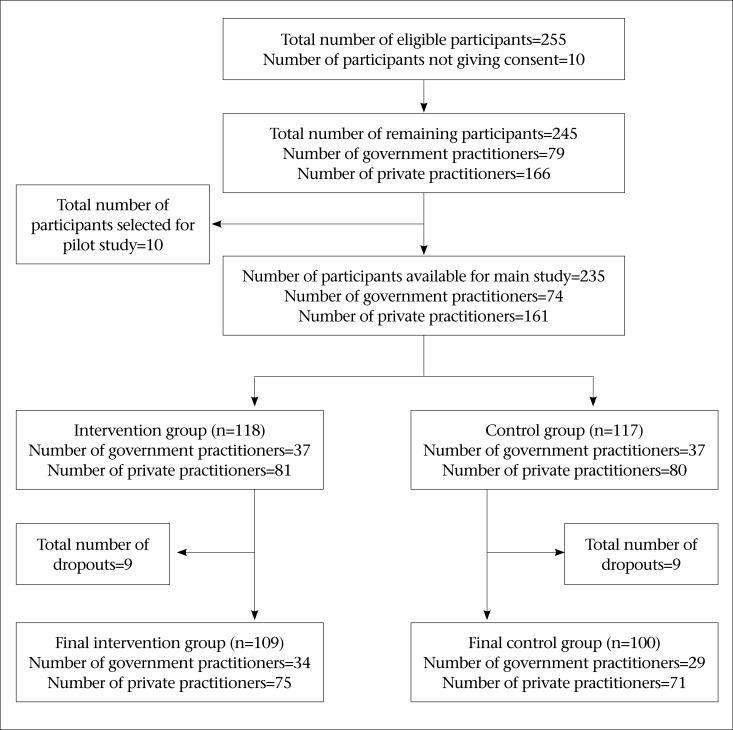
Sampling method (N=209)

The study population comprised healthcare providers in Banke district, who had legal right to prescribe medicines and were working in primary healthcare settings and pharmacies. They included persons with the qualification of health assistants (HAs), community medical assistants (CMAs), auxiliary health workers (AHWs), and assistant nursing midwives (ANMs). There were altogether 255 eligible participants in Banke district but, after omitting those who did not give their consent, they were involved in pilot study of this research, and those who dropped out due to various reasons, only 209 participants were included in all analyses in this research. The main reasons for drop-out were migration, unavailability of participants during data collection, and changing and closing of their clinics. The details are shown in Figure 1.

### Inclusion criteria

Healthcare providers working in the government sub-health posts (SHPs), health posts (HPs) and primary healthcare centres (PHCCs) were included in the study, and those eligible healthcare providers who were practising in a pharmacy registered with the Department of Drug Administration were also the part of the study.

### Exclusion criteria

The study excluded the following individuals:

(a) People working in pharmacies but do not have legal right to prescribe the medicines
(b) Practitioners working in their home-based setting (neither government institution nor registered pharmacies)
(c) People working in both private and government settings were taken as government practitioners only. The interventions were carried out in government setting, and data from them were also collected from the same setting
(d) Ten practitioners who were participants in the pilot study
(e) Subjects who were not interested and refused to participate in the study.

### Study sample

Stratified randomized sampling was done. The population was divided into two different strata—intervention and control group. The whole method of sampling is shown in Figure 1.

### Method of randomization

In the case of government healthcare providers, the list of healthcare providers working in SHPs, HPs, and PHCs were obtained from the district public health office. The names given by the office were already assigned in a numerical order. All health workers having odd numbers were treated as intervention group, and those with even numbers were treated as control group.

Similarly, the list of registered pharmacies within Banke district was obtained from the Department of Drug Administration, Mid-western Regional Office. The list contained the names of pharmacies and names of persons under whom the pharmacies were registered. The researchers took the list and conducted a cross-sectional survey to find out the eligible prescribers from the list. The names of the eligible prescribers were arranged in alphabetical order and were numbered. All the names falling under odd numbers were treated as intervention group and the remaining as control group.

### Study tools

The following study tools were used for this part of the study:

#### Data-collection form to collect information on prescribed medicines

A form was developed to collect information on the names of medicines and total cost of medicines prescribed to the simulated client at baseline, first, and second follow-up phase of the study.

#### Academic detailers

There were altogether two academic detailers. The first one was a pharmacist with the B.Pharm degree, and the second one was a medical officer with a graduate-level medical education (MBBS). Both the academic detailers were from the local place and were good in English, Nepalese, and local languages of the district. The first academic detailer was trained in KIST Medical College, Lalitpur, Nepal. KIST Medical College was the only medical college in Nepal conducting regular academic sessions to the clinicians working in the teaching hospital of the same college (14). The first academic detailer conducted the pilot study of this research and, using the experience gained, he trained the second academic detailer. Both the academic detailers provided demonstration detailing on the given topic to various levels of healthcare providers and healthcare students before going to the field.

#### Simulated clients

The simulated clients comprised community medical assistants as students. The observation technique for simulated clients was adopted from the method mentioned by Hardon *et al.* (15). The simulated clients were trained about the symptoms of acute diarrhoea without dehydration, and were sent with a child between ages of 3 and 4 years to the healthcare providers to collect information on prescribed medicines. The child was also simulated; he did not have any diarrhoeal episodes. They were given data-collection form to collect information on prescribed medicines and were asked to fill these in immediately after each visit.

### Ethical considerations

Ethical approval was taken from the Institutional Review Board, Nepalgunj Medical College and Teaching Hospital prior to conducting the experiment. Verbal consents were taken from all participants via telephone calls before starting the data collection. The participants were given the description about objective of the study; the participants were also told they would attend visit by a simulated client three times during the study period. Confidentiality of the data was also assured to all the participants by using coding method to record their responses.

### Operational modality

**Figure 2.**
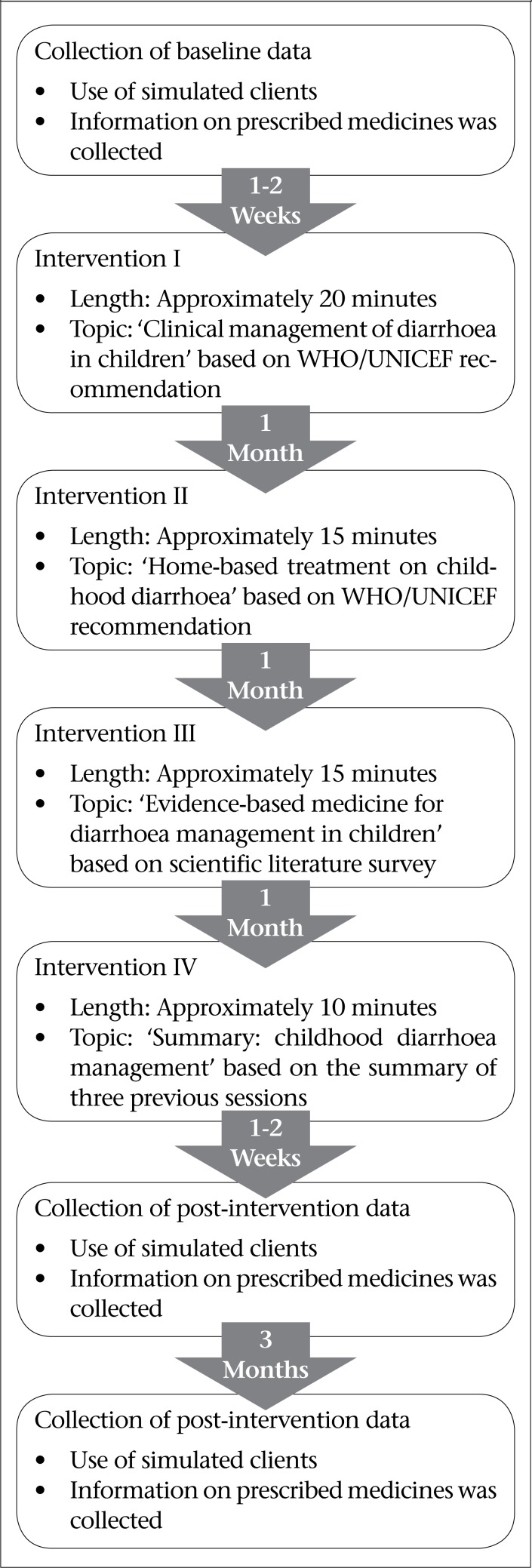
Flowchart showing operational modality of research

The phases in the study were: collection of pre-intervention data, intervention in the form of academic detailing and collection of post-intervention data soon after four series of academic detailing sessions, and post-intervention data collection once again after three months of wash period. The details of operational modality are shown in Figure 2.

### Collection of baseline data

Baseline data on prescribing patterns in acute diarrhoea without dehydration were obtained by sending simulated clients. Then the medicines prescribed were entered in the data-collection from.

### Interventions

Intervention was carried out in the form of academic detailing. Four consecutive academic detailing sessions were conducted for the intervention group for three months with an interval of one month between sessions. The academic detailing sessions were conducted by trained academic detailers. Academic detailing sessions were conducted as per the technique suggested by Soumerai and Avorn (6). The process was slightly modified according to the local setting and expertise.

#### Intervention I

Academic detailer spent around 20 minutes with each prescriber in this intervention. The topic was “Clinical management of diarrhoea in children” based on the WHO/UNICEF recommendation. The emphasis was on promoting the prescription of ORS and zinc to the diarrhoeal child. The intervention was also intended to decrease the use of antiprotozoals, antimicrobials, medicines acting on GI motility, use of vitamins and enzyme preparations in cases of acute diarrhoea. The session detailed the dosage regimens of ORS and zinc at normal dose—the situation when patients should receive fluid via intravenous and nasogastric route.

#### Intervention II

Academic detailer spent around 15 minutes with each participant. The topic was “Home-based treatment of childhood diarrhoea” based on the WHO/UNICEF recommendation. The participants were trained regarding home-based alternatives to ORS, and also about the recommended foods and contraindicated drinks during this intervention. Importance of breastfeeding during diarrhoea was also mentioned. Finally, participants were told to counsel caretakers of every participants regarding home-based treatment in childhood diarrhoea.

#### Intervention III

This was done one month after Intervention II. Academic detailer spent around 15 minutes with each prescriber. The topic was “Evidence-based medicines for diarrhoea management in children” based on our scientific literature survey. Various sources of literature were surveyed to find out the importance of ORS and zinc in childhood diarrhoea; literature regarding non-evidence-based use of other medicines during diarrhoea was also collected to develop the intervention tool. With the help of that intervention tool, real scientific reason behind prescribing ORS and zinc was discussed with participants. They were also told about the reason why antiamoebics, antimicrobials, medicines affecting GI motility, multivitamins, and enzyme preparations may not be necessary in most diarrhoeal cases. Data on mortality and morbidity due to childhood diarrhoea was also presented to them to sensitize them regarding childhood diarrhoeal problem.

#### Intervention IV

This was done one month after Intervention III, and the academic detailer spent around 10 minutes with each prescriber. The topic was based upon the summary of all three previous interventions. All the findings from Intervention I, Intervention II, and Intervention III were summarized. The key points from the previous interventions were focused.

### Collection of post-intervention data

Post-intervention data were collected in two phases—first follow-up and second follow-up. The first follow-up data were collected 10-15 days after the last intervention (Intervention IV). Data on prescribing patterns in acute diarrhoea were collected with the help of simulated clients. Medicines prescribed to the simulated clients were entered in the data-collection form. Data on the second follow-up were collected three months after the collection of the first follow-up data to find out sustainability of the impact of intervention. The type of data collected and tools and method used were similar to those used for baseline and the first follow-up.

### Evaluation of adherence to diarrhoea treatment guidelines

Simulated clients used in the study were trained on the symptoms of acute diarrhoea without dehydration. Only the participants prescribing evidence-based medicines were considered to be adherent to diarrhoea treatment guidelines. The evidence-based regimen for this condition consisted of only ORS and zinc supplementation. The prescription containing more than these two drugs was considered non-evidence-based. The prescriptions containing either ORS or zinc were also not considered evidence-based prescription.

### Method of data analyses

Microsoft Excel 2003 and Statistical Package for Social Sciences (SPSS) for Windows (version 16) were used for statistical analyses. Frequency, mean, standard deviation, median, and interquartile range were used for descriptive purposes. The normality of the data was calculated by Kolmogorov-Smirnov test and was found to be non-linear or non-normal. Therefore, non-parametric tests, like chi-square test, Mann-Whitney U-test and Friedman test were used throughout the study where appropriate. A priori significant level of 0.05 was used in all analyses.

## RESULTS

### Demography of the participants

From among all participants, 209 were included in the analytical part of the study. The mean±SD age of the participants was 34.1±8.3 years. The median age of participants was 32 years [Interquartile range (IQR)=26-32 years]. The mean and median of the length of experience in treating patients were 7.3±3.3 and 8 years respectively (IQR=4-13 years). The control group and the intervention group of participants were not significantly different in terms of gender, age, type of organization, qualification, experience, and number of daily patient-visits. Details on demography are illustrated in Table 1.

### Prescribing pattern of the participants

The baseline data revealed under-prescription of ORS and zinc by the participants of the study. At baseline, there was no significant difference between the control and the intervention group in terms of prescribing ORS and zinc. However, data from the first follow-up and second follow-up revealed that there was significant increase in the prescription of ORS and zinc by participants in the intervention group compared to the control group. Antimicrobials, like metronidazole, fixed-dose combination of metronidazole and diloxanide furoate, ciprofloxacin, norfloxacin, and nalidixic acid were prescribed at baseline by some participants. After intervention, there was a marked decrease in the number of participants prescribing metronidazole, fixed-dose combination of metronidazole and diloxanide furoate. However, there was no significant difference between the control and the intervention group in terms of prescribing ciprofloxacin, norfloxacin, and nalidixic acid. Data from the study also revealed a reduction in the prescription of multivitamins and enzyme preparations by the participants in the intervention group compared to the control group during the first and the second follow-up. Another analysis was done to see the changes in the prescribing pattern among participants from baseline to the first and the second follow-up. Data revealed that there was a significant increase in the number of participants prescribing ORS among both control and intervention group from baseline to the first and the second follow-up. Similarly, there was also an increase in the number of participants prescribing zinc by participants of the intervention group from baseline to the first and the second follow-up. However, such changes were not observed among participants in the control group. A marked decrease in the number of participants prescribing metronidazole among both control and intervention group was observed during the first and the second follow-up compared to baseline among both control and intervention group. In the case of fixed-dose combination of metronidazole and diloxanide furoate, multivitamins, and enzyme preparations, the marked decreased was only seen among participants in the intervention group from baseline to the first and the second follow-up. However, such changes were not seen among participants in the control group. There was no marked change in the number of participants prescribing ciprofloxacin, norfloxacin, and nalidixic acid in both control and intervention group from baseline to the first and the second follow-up phase of the study. Details of the change in the prescribing pattern among participants from baseline to the first and the second follow-up phase of the study for both control and intervention group are given in Table 2.

**Table 1.** Demographic characteristics of participants

Characteristics	Number of participants		
	Control group (n=100)	Intervention group (n=109)	p value
Gender			
Male	66	72	0.993
Female	34	37	
Age			
21-<31 years	49	45	0.572
31-<41 years	38	46	
41-50 years	11	13	
>50 years	2	3	
Type of organization			
Government	29	34	0.730
Private	71	75	
Location			
Urban	48	53	0.928
Rural	52	56	
Qualification			
AHW	44	46	0.847
CMA	6	6	
HA	36	45	
NM	14	12	
Experience (in years)			
<6 years	31	35	0.082
6-<11 years	40	37	
11-<16 years	11	22	
16-20 years	11	8	
>20 years	7	7	
Number of daily patient-visits			
<11	6	4	0.915
11-20	13	12	
21-30	35	43	
31-40	13	17	
41-50	11	10	
>50	22	23	

p value (two-sided) was calculated by chi-square test; AHW=Auxiliary health worker; ANM=Auxiliary nurse-midwife CMA=Community medical assistant; HA=Health assistant

#### Percentage of participants adhering to the diarrhoea treatment guidelines

At baseline, less than 10% of the participants were adhering to the diarrhoea treatment guidelines, and there was no significant difference between the control and the intervention group in terms of percentage of participants adhering to the diarrhoea treatment guidelines. However, there was a significant increase in the number of participants adhering to the diarrhoea treatment guidelines among the intervention group compared to the control group. There was a remarkable increase in the number of participants among both control and intervention group adhering to the diarrhoea treatment guidelines from baseline to the first and the second follow-up phase of the study. Data on change in the percentage of participants adhering to the diarrhoea treatment guidelines from baseline to the first and the second follow-up phase of the study are given in Table 3.

#### Cost of medicines prescribed by participants working in private setting

The cost of medicines prescribed by participants working in private setting was analyzed at baseline, the first follow-up, and the second follow-up phase of the study. Among 146 private practitioners participating in this study, 71 were in the control group and 75 in the intervention group. At baseline, there was no significant difference between the control and the intervention group in terms of prescription cost. However, after intervention during the first and the second follow-up, there was a significant reduction in the prescription cost in the intervention group compared to the control group. Data also showed that there was a statistically-significant decrease in the cost of medicines prescribed by participants in the intervention group from baseline to the first and the second follow-up phase of the study. However, such changes were not seen among participants in the control group. Data on changes on cost of the prescribed medicines at various stages of the study are presented in Table 4.

## DISCUSSION

Childhood diarrhoea is one of the major causes of mortality and morbidity in Nepal (16). Studies done in many countries reported diarrhoea management as one of the areas where we can find many cases of non-evidence-based prescriptions, including unnecessary prescription of antimicrobials, medicines affecting gastric motility, multivitamins, and enzyme preparations (17-19). The magnitude of non-evidence-based prescription will be much greater in the presence of other confounding factors that negatively influence prescribing patterns. A survey done in six major cities of Nepal reported that the pharmacists working in the community settings lacked proper books and had limited access to reliable unbiased source of information on drugs (20,21). An article suggested that, with increase in the number of pharmaceutical companies, clinicians in Nepal are in high risk of receiving biased information from their pharmaceutical representatives (22). Limited access to the unbiased source of information on medicines, coupled with the incidence of receiving biased information from the pharmaceutical companies, may put primary healthcare providers in Banke district at higher risk of prescribing wrongly. Therefore, four academic detailing sessions on childhood diarrhoea management were conducted for the primary healthcare providers in Banke district of Nepal. Of 209 participants, academic detailing sessions were provided to 109 participants (intervention group) while 100 participants (control group) were not provided with academic detailing sessions. The demographic data showed that participants in the control and intervention groups were similar in terms of gender and age distribution. They were also homogeneous in terms of the type of organization where they were working (private or government), qualification, and the estimated number of patients visiting their clinic daily.

**Table 2.** Change in prescribing pattern among participants at various phases of the study

Treatment	Percentage of participants		p value
	Control group (n=100)	Intervention group (n=109)	
ORS			
Baseline	60	57.8	0.747
First follow-up	72	91.7	<0.001*
Second follow-up	79	95.4	<0.001*
p value	<0.001*	<0.001*	
Zinc			
Baseline	32	31.2	0.900
First follow-up	62	87.2	<0.001*
Second follow-up	76	96.7	<0.001*
p value	<0.001*	<0.001*	
Metronidazole			
Baseline	56.0	54.1	0.786
First follow-up	44.0	7.3	<0.001*
Second follow-up	20.0	4.6	0.006*
p value	0.014*	<0.001*	
Metronidazole+Diloxanide Furoate Combination			
Baseline	18.0	19.2	0.814
First follow-up	20.0	6.4	0.003*
Second follow-up	18.0	6.4	<0.001*
p value	0.916	0.002*	
Ciprofloxacin			
Baseline	6.0	1.2	0.117
First follow-up	5.0	1.8	0.204
Second follow-up	3.0	3.7	0.788
p value	0.248	0.115	
Norfloxacin			
Baseline	3.0	1.8	0.582
First follow-up	2.0	1.8	0.972
Second follow-up	3.0	4.6	0.550
p value	0.346	0.114	
Nalidixic acid			
Baseline	3.0	1.8	0.582
First follow-up	5.0	1.8	0.204
Second follow-up	5.0	1.8	0.204
p value	0.548	0.692	
Multivitamins			
Baseline	19.0	19.3	0.961
First follow-up	16.0	5.5	0.014*
Second follow-up	21.0	0659	0.002*
p value	0.001*	6.4	
Enzyme preparations			
Baseline	11.0	12.8	0.682
First follow-up	14.0	5.5	0.037*
Second follow-up	16.0	0.810	4.6
p value	0.046*	0.032*

p value (two-sided) was calculated by chi-square test;

^*^p value significant at α = 0.05

Oral rehydration solution (ORS) is considered the first-line treatment for any diarrhoeal child (23). However, our finding suggested that only about half of the participants prescribed ORS to the simulated clients, indicating that there was under-prescription of ORS among the primary healthcare providers of Banke district of Nepal. Finding of the under-use of ORS was in contrast to annual health report of Nepal, which highlighted 99% use of ORS by diarrhoeal children in Banke district of Nepal (16). However, after four successive evidence-based academic detailing sessions on childhood diarrhoea and its management, there was a marked increase in prescribing ORS among participants in the intervention group compared to the control group during the first and the second follow-up phase of the study. These improvements were higher in magnitude compared to findings of a meta-analysis on academic detailing (7). This improvement can be attributed to the format of this academic detailing programme; four consecutive academic detailing sessions on the same topic were done, which was uncommon with regard to published studies (7). Similarly, other studies conducted in Kenya and Indonesia also reported a significant increase in the use of ORS following academic detailing sessions for pharmacists (4).

A meta-analysis showed that the incidence, severity, and episodes of diarrhoea are significantly reduced following administration of zinc supplementation with ORS (24). Therefore, World Health Organization and United Nations Children's Fund jointly recommended ORS and zinc therapy to manage diarrhoea in children (23). However, baseline data of this study suggested that a very few participants prescribed zinc to their simulated clients. This can be attributed to the fact that zinc was just newly introduced in Nepalese market (25). However, after the intervention, there was a statistically-significant increase in the number of participants prescribing zinc among both control and intervention group. This might be due to the nationwide campaign to promote the use of ORS and zinc (25). However, a significant difference between the control and the intervention group during both first and second follow-up shows that participants in the intervention group were prescribing zinc more compared to the control group. This shows that academic detailing can also be used as a tool to provide information on new medicine to the clinicians. The effectiveness of academic detailing, as a possible source of information on medicine in Nepal, has been already discussed elsewhere (26).

**Table 3.** Change in percentage of participants adhering to the diarrhoea treatment guidelines during different phases of the study

Phase	Percentage of participants		p value
	Control group (n=100)	Intervention group (n=109)	
Baseline	6.0	8.3	0.528
First follow-up	16.0	65.1	<0.001*
Second follow-up	19.0	69.7	<0.001*
p value	0.011*	<0.001*	

p value (two-sided) was calculated by chi-square test;

^*^p value significant at α = 0.05

**Table 4.** Change in the cost prescribed medicines at various stages of the study

Phase	Total cost of prescription in Nepalese Rupees		p value
	Control group (n=71)	Intervention group (n=75)	
Baseline			
Mean±SD	65.6±33.0	67.1±37.2	0.994
Median (IQR)	68.0 (40-88)	64.0 (38-89)	
First follow-up			
Mean±SD	64.9±33.0	54.7±29.6	0.034*
Median (IQR)	65.0 (38-90)	44.0 (38-66.5)	
Second follow-up			
Mean±SD	65.8±33.0	52.6±28.5	
Median (IQR)	62.0 (38-88)	44 0 (38-58)	0.007*
p value	0.894	0.098*

p value (two-sided) was calculated by Mann-Whitney U-test in horizontal axis and by Friedman test in vertical axis of this table;

^*^p value significant at α = 0.05

The baseline data of the study also showed prescriptions of a large number of other medicines, like-metronidazole and fixed-dose combination of metronidazole and diloxanide furoate to the simulated clients having acute diarrhoea without dehydration. Such antiprotozoals are not required for the patients unless the diarrhoea is infectious (23). Many studies reported the intensive use of antimicrobials and other unnecessary medicines even during non-infective diarrhoea (17,19,27,28). However, in this study, only a few of the participants prescribed such antimicrobials. Multivitamins supplementation and enzyme preparations are usually advised in case of malnutrition (28). During simple acute diarrhoea, the prescription of such preparations only increases the economic burden for the patients (24). The academic detailing significantly improved the prescribing pattern of the participants. There was a significant reduction in the number of participants prescribing different antimicrobials, multivitamins, and enzyme preparations among the intervention group compared to the control group. For increase in the number of participants prescribing useful medicines and decrease in the number of participants prescribing unnecessary medicines, it is evident that academic detailing can promote rational prescribing, justifying the first two hypotheses of this study. The number of participants adhering to the diarrhoea treatment guidelines also significantly increased among the intervention group compared to those in the control group during the first and the second follow-up phase of the study, which was in agreement with the third hypotheses set prior to the study. After academic detailing, there was also a significant decrease in the cost of medicines prescribed to simulated clients by participants in the intervention group compared to participants in the control group. These all suggested that academic detailing was feasible in resource-limited setting and was able to improve the prescribing behaviour of the participants and their adherence to the childhood diarrhoea treatment guidelines, and decrease the cost of medicines prescribed to the patients. These findings were close to the findings of previous studies that evaluated the impact of academic detailing in developed countries (1,29-32). Therefore, academic detailing can even be practised in resource-limited countries, like Nepal. The sessions on information regarding newer medicines in the market, reporting of adverse drug reaction, rationality of fixed-dose combination of drugs, rational use of antimicrobials, and proper use of non-steroidal anti-inflammatory drugs, may be some important areas for academic detailing. Academic detailing has an ample scope in Nepal and can contribute a lot to the clinicians who have very limited access to the source of unbiased information on medicines (11,26).

### Limitations

This study was done on the primary healthcare providers in Banke district of Nepal. It may be difficult to generalize these findings for other districts of Nepal. The primary healthcare providers working on their home-based settings (neither government centre nor private clinic) were not included in this study. In analyses, drop-out cases were not considered. Prescribing behaviour was difficult to evaluate with the simulated clients; so, only prescribing patterns were studied. The study did not evaluate the effect of confounding factors, like role of media, pharmaceutical representatives, and other forms of training which participants might have taken during study period. In the economic evaluation part of the study, only private participants were considered because medicines at government primary healthcare facilities are provided free of charge to the patients. This study could not evaluate the actual economic benefit of academic detailing; it did not consider the investment in academic detailers, academic detailing tools, travel expenses, and other miscellaneous expenses.

### Conclusions

The present study successfully evaluated the impact of academic detailing programme on prescribing pattern, adherence of participants to childhood diarrhoea treatment guidelines and prescription cost among the primary healthcare providers in Banke district of Nepal. Baseline data showed under-use of ORS and zinc, unnecessary prescribing of some antimicrobials, vitamins, and enzyme preparations. It showed that participants in the control group adhered to the diarrhoea treatment guidelines. However, academic detailing increased the use of ORS and zinc and decreased the prescribing of unnecessary antimicrobials, vitamins, and enzyme preparations. Academic detailing also decreased the cost of prescription for the patients.

Academic detailing can be used as a good means of information on medicines for the primary healthcare providers in developing countries, like Nepal. The programme can successfully improve the prescribing pattern of the primary healthcare providers. Although this study shows less magnitude of cost reduction for treatment, it may be very significant for the healthcare financing bodies at policy level. Therefore, this type of programme should be taken up by universities, social welfare organizations, professional societies, policy-makers, and relevant stakeholders in order to improve rational use of medicines in the community.

## ACKNOWLEDGEMENTS

Authors would like to thank all participants who participated in this study. Authors are indebted to all the resource persons and data collectors associated with the research and would like to thank Nepalgunj Medical College and Teaching Hospital for granting permission to conduct this study. Finally, a special thank goes to the Universiti Sains Malaysia, Penang, Malaysia, for partially funding this research.
